# Conversion Surgery for Stage IV Gastric Cancer

**DOI:** 10.3389/fonc.2019.01158

**Published:** 2019-11-07

**Authors:** Fei Zhang, Xuanzhang Huang, Yongxi Song, Peng Gao, Cen Zhou, Zhexu Guo, Jinxin Shi, Zhonghua Wu, Zhenning Wang

**Affiliations:** Key Laboratory of Precision Diagnosis and Treatment of Gastrointestinal Tumors, Ministry of Education, Department of Surgical Oncology and General Surgery, The First Affiliated Hospital of China Medical University, Shenyang, China

**Keywords:** conversion surgery, conversion therapy, metastatic gastric cancer, unresectable gastric cancer, combined chemotherapy, stage IV gastric cancer

## Abstract

The prognosis of stage IV gastric cancer (GC) is poor, with palliative chemotherapy remaining the main therapeutic option. Studies increasingly indicate that patients with unresectable stage IV GC, who undergo gastrectomy with radical intention after responding to several regimens of combined chemotherapy, can achieve good survival outcomes. Thus, surgery aiming at radical resection for unresectable stage IV GC after combined chemotherapy has received increasing attention in recent years. This novel therapeutic strategy was defined as conversion surgery in patients with unresectable stage IV GC and it can associate with significant improved survival when R0 resection can be achieved. Despite the recent advances in conversion surgery for patients with unresectable stage IV GC, selection criteria for combination chemotherapy regimens, indications for conversion surgery, optimal timing to surgery, and postoperative chemotherapy all remain controversial. This article reviews the current state of conversion surgery for unresectable stage IV GC.

## Introduction

Despite early screening and improved intensive therapy, gastric cancer (GC) remains to be the fifth most common cancer and third most common cause of cancer-related deaths worldwide, leading to increased health care burden (1, 2). The prognosis for patients with stage IV gastric cancer is poor, and palliative chemotherapy remains the main therapeutic approach for this cohort (3, 4). Despite recent developments in chemotherapy, the median overall survival (OS) of stage IV GC patients remains to be 9–11 months (5, 6). For unresectable stage IV GC patients, only surgeries to relieve symptoms, such as a palliative resection or bypass operations, are commonly considered (7–9). The possibility of additional survival benefits achieved by chemotherapy following palliative surgery has been controversial. Recently, the REGATTA trial randomized 175 stage IV GC patients with a single incurable factor (liver metastases, peritoneal metastases, or para-aortic lymph node metastases) to chemotherapy alone or to initial gastrectomy followed by chemotherapy, but the surgery-first approach failed to show improvements in survival (10). Even with the greater advances achieved by adding a targeted monoclonal antibody to conventional chemotherapy (11–13), the prognosis for unresectable stage IV GC is still unsatisfactory. Thus, novel therapeutic strategies are required for treating unresectable stage IV GC patients.

Several combined S-1 based chemotherapy regimens may allow for conversion of initially unresectable GC to resectable cancer in clinical trials, and additional surgery following this chemotherapy was associated with long-term survival in selected patients (14–16). These advances raise new clinical issues in the treatment of unresectable stage IV GC patients. In some patients, incurable factors apparently disappear or are well-controlled during chemotherapy. For such patients, surgery with curable intent may be possible. Previous reviews investigating the effects of surgery for unresectable stage IV GC patients after chemotherapy also indicate that surgery with curable intention after chemotherapy is associated with prolonged survival in selected patients with a single incurable factor (17, 18), such as liver metastases, peritoneal metastases, or para-aortic lymph node metastases (19, 20). Thus, “conversion surgery” is defined as a surgical treatment aiming at a curable intention after tumors initially deemed technically or oncologically unresectable or only marginally resectable respond to chemotherapy (21, 22). Notably, conversion surgery refers to a radical cure and is different from palliative surgery or other terms concerning surgical resection for advanced incurable tumors such as “salvage,” “adjuvant,” or “secondary” surgery (23–27). Furthermore, there is no consensus on a clear border between curative surgery scheduled after neoadjuvant chemotherapy and conversion surgery (28, 29). Neoadjuvant chemotherapy can be used for initially resectable advanced GC to reduce tumor size and eradicate micrometastases to improve survival (30, 31), whereas chemotherapy for conversion surgery is used for unresectable advanced GC patients (18, 21).

Developments in conversion surgery have improved life expectancy in patients with incurable advanced GC, attracting increasing attention to conversion surgery for unresectable advanced GC in recent years (18, 32–34). However, selection criteria for combination chemotherapy regimens, indications for conversion surgery, optimal time to surgery, and postoperative chemotherapy all remain controversial. Therefore, we summarize the current state of the field regarding conversion surgery for stage IV GC in this review.

## Search Strategy and Selection Criteria

We searched PubMed for articles published in English from 1997 to 2019 using the search terms “gastric cancer”, “conversion therapy,” “conversion surgery,” “stage IV gastric cancer,” “incurable advanced GC,” and “unresectable advanced GC.” No other parameters were applied. Case reports were excluded. Ultimately, 20 articles were included (shown in Table 1).

**Table 1:** Unresectable characteristics and conversion surgical treatments.

**Year**	**References**	**Unresectable criteria**					**Chemotherapy**	**Surgery type**	**≥D2**	Conversion surgery (%)^*^	R0 *n* (%)^**^
		**T4**	**P1**	**H1**	**PAN/N3**	**Other**					
1997	Nakajima et al. (35)	8 (27%)	9 (30%)	11 (37%)	23 (77%)	3 (10%)	FLEP	NS	NS	19/30 (63.33%)	9 (47%)
2002	Yano et al. (36)	12 (35%)	26 (76%)	4 (12%)	10 (3.4%)	1 (3.4%)	FEMTXP or THP-FLPM	NS	NS	14/34 (41.17%)	8 (57%)
2012	Satoh et al. (15)	−	24 (49%)	3 (6%)	7 (14%)	17 (33%)	S1+Cisplatin	TG (58.0%) DG (21.5%)	82%	44/51(86.27%)	26 (59%)
2012	Kanda et al. (16)	9 (32%)	7 (25%)	4 (14.3%)	15 (54%)	−	S1 + Cisplatin or Paclitaxel or Irinotecan	TG (42.89%) DG (57.1%)	96.30%	28/31 (90.32%)	26 (93%)
2013	Han et al. (37)	−	7 (14%)	5 (10%)	15 (29.4%)	7 (14%)	5-FU ± Platinum or Taxane ± 5-FU ± Platinum	NS	NS	34/34 (100%)	26 (76%)
2014	Kim et al. (38)	−	43 (100%)	−	−	−	5-FU + Cisplatin or S1 + Cisplatin	TG (72.2%) DG (27.7%)	100%	18/43 (41.86%)	10 (55%)
2014	Saito et al. (39)	9 (10.22%)	26 (29.54%)	7 (7.95%)	21 (23.86%)	7 (7.95%)	S-1 + cisplatin	TG (38.4%) DG (61.6%)	100%	59/88 (67.04%)	13 (22%)
2015	Fukuchi et al. (22)	6 (15%)	11 (28%)	5 (13%)	−	29 (73%)	S1 + Cisplatin or S1 + Paclitaxel	TG (72.5%) DG (27.5%)	NS	40/151 (26.49%)	32 (80%)
2015	Kinoshita et al. (40)	−	15 (26%)	18 (32%)	23 (40%)	2 (3.5%)	DCS	TG (64.7%) DG (26.5%)	50%	34/57 (59.64%)	27 (79%)
2017	Sato et al. (41)	14 (14%)	33 (33%)	29 (29%)	61 (61%)	11 (11%)	DCS Iline, CPT-11 II line	TG (84.8%) DG (12.1%)	100%	33/100 (33%)	28 (85%)
2017	Mieno et al. (42)	8 (25.8%)	8 (25.8%)	5 (16%)	18 (58%)	−	DCS + DS	TG (74.2%) DG (22.6%)	77%	31	23 (74%)
2017	Uemura (43)	6 (13.9%)	16 (37.2%)	14 (32.6%)	22 (51.2%)	4 (9.3%)	Modified DCS	NS	100%	43/49 (87.75%)	15 (35%)
2017	Einama et al. (44)	1 (10%)	3 (30%)	1 (10%)	4 (40%)	1 (10%)	S1 + CDDP or DOC	TG (40%) DG (30%)	100%	10	10 (100%)
2017	Maeda et al. (45)	−	−	3 (37.5%)	8 (100%)	−	Modified DCX	NS	100%	3/8 (37.5%)	3 (100%)
2017	Yamaguchi et al. (46)	−	35 (41%)	−	37 (44%)	34 (40%)	DCS or S1 or S1 + Cisplatin or S1 + Taxane	TG (82.1%) DG (17.9%)	NS	84/259 (32.43%)	43 (51%)
2017	AIO-FLOT3 (29)	13 (21.8%)	4 (6.7%)	11 (18.3%)	36 (60.1%)	2 (3.3%)	FLOT	NS	NS	36/60 (60%)	29 (80%)
2018	Morgagni et al. (47)	8 (36.36%)	2 (9.09%)	2 (9.09%)	11 (50%)	−	Epirubicin + Cisplatinum + 5-FU or Oxaliplatin + 5-FU or Docetaxel + Oxaliplatin + 5-FU or Other	TG (72.7%) DG (22.7%)	91.9%	33/57 (57.89%)	22 (67%)
2018	Beom et al. (32)	2 (2.0%)	33 (32.7%)	11 (10.9%)	35 (34.7%)	20 (19.8%)	Platinum + 5-FU or Taxane + 5-FU or Platinum + Taxane + 5-FU or Taxane + Platinum or Others	TG (56.4%) DG (43.6%)	75.2%	101	57 (56%)
2019	Solaini et al. (48)	−	38 (84.4%)	4 (8.8%)	3 (6.6%)	−	Cisplantin + 5-FU or Epirubicin + Cisplatinum + 5-FU or Docetaxel + Oxaliplatin + 5-FU or Other	TG (73.3%) DG (26.7%)	91.1%	45	30 (67%)
2019	Li et al. (49)	−	8 (9.8%)	10 (12.2%)	60 (74.1%)	3 (3.7%)	Oxaliplatin + 5-FU (Capecitabne or S-1) or Oxaliplatin + 5-FU + Docetaxel/Anthracyclines	NS	NS	81/414 (19.5%)	66 (81.4%)

*P1, Peritoneal carcinomatosis; H1, Hepatic metastases; PAN, Para-aortic node metastases; TG, Total gastrectomy; DG, Distal gastrectomy; DCS/DS: Docetaxel-Cisplatin-S1/Docetaxel-Cisplatin; FEMTXP: Fluorouracil, epirubicin, methotrexate, cisplatin; THP-FLPM: Pirarubicin, 5-FU, Leucovorin, Cisplatin, mitomycin C; FLEP: 5-FU + Leucovorin + Etoposide; CDDP: Cisplatin; DOC: Docetaxel; FLOT: fluorouracil, leucovorin, oxaliplatin, and docetaxel*;

* *Conversion surgery rate: (conversion surgery number) / population × 100%*;

** *R0 resection rate: (R0 resection number) / (conversion surgery number) × 100%; NS: Not specified*.

## Conversion Surgery of Peritoneal Dissemination

Peritoneal metastases (PM), or peritoneal carcinomatosis, is the most common type of metastasis in stage IV GC with poor prognosis (38, 50, 51). Although GC patients with PM undergo combined intensive chemotherapy, the prognosis for this cohort was still unsatisfactory due to their relative resistance to systemic chemotherapy and low drug delivery into the abdominal cavity (35, 36). Developments in S-1 based chemotherapeutic regimens (S-1 plus cisplatin, SP; docetaxel plus cisplatin and S-1, DCS) for advanced GC patients (52–55) resulted in improved overall survival (OS) rate for advanced GC patients with PM. Thus, these advances in chemotherapy are expected to improve survival in unresectable stage IV GC patients with PM. A phase II trial of preoperative S-1 plus cisplatin (SP, oral S-1 plus intravenous cisplatin) chemotherapy, followed by gastrectomy with curable intention in unresectable stage IV GC patients with PM, showed a high response rate to SP with a longer OS over chemotherapy alone. Although most of the eligible patients in this trial had PM, R0 resection was still achieved in 51% of patients after preoperative SP chemotherapy, suggesting that controlling peritoneal dissemination is extremely important in conversion surgery for this cohort (15). Similarly, a trial of SP induction chemotherapy, followed by curative resection in unresectable stage IV GC patients with PM, showed a good response to SP chemotherapy followed by R0 resection with a high median survival time (MST) relative to chemotherapy alone (39). Despite advances in intravenous chemotherapy for unresectable stage IV GC patients with PM (22, 38–40, 55), drug delivery into the abdominal cavity remained low and sustained intraperitineal concentrations were still relatively poor with limited controlled efficacy (56).

Since intraperitoneal (IP) delivery of chemotherapy can attain higher drug exposure in the peritoneal cavity with reduced systemic toxicity (57), intraperitoneal administration of paclitaxel can provide sustained high local concentrations to increase its antitumor effects in GC patients with PM (58). Although promising results have been achieved for combination chemotherapy of IP paclitaxel with S-1 for patients with unresectable GC and PM, yielding a MST of 17.6 months and a 1-year OS of 77.1%(59), salvage gastrectomy on advanced GC patients with PM after disappearance or apparent shrinkage of PM yielded a MST of 26.4 months and a 1-year OS of 82%(60), indicating that conversion surgery may be considered in the cohort with a favorable response after IP paclitaxel plus systemic chemotherapy (50). A single-arm phase II study of conversion surgery following eight cycles of IP paclitaxel with systemic oxaliplatin and capecitabine (XELOX) in unresectable GC patients with PM and/or positive peritoneal washing cytology showed that six patients who underwent conversion gastrectomy, after a favorable response rate to combined XELOX and IP paclitaxel experienced a MST of 21.6 months, compared to patients receiving systemic chemotherapy alone in other trials who had MST of 3.1–10.6 months (50). Additionally, a recent meta-analysis indicated that there are survival benefits associated with hyperthermic intraperitoneal chemotherapy (HIPEC), delivering a high drug concentration for advanced GC patients with PM involvement compared with systemic chemotherapy alone (61). A cohort study of conversion surgery after HIPEC, plus chemotherapy in a small cohort of stage IV GC patients with PM, showed good long-term outcomes, suggesting that combination HIPEC may represent a useful and feasible technique to improve survival in GC patients with PM undergoing conversion surgery (47). In a GIRCG retrospective cohort study, 23 unresectable stage IV GC patients with PM received conversion gastrectomy after HIPEC plus chemotherapy, with a conversion rate of 60.5%(48).

A phase III trial was conducted (PHOENIX-GC), with the IP arm showing a better response in the amount of ascites and a high negative conversion rate of 78% for peritoneal cytology, further supporting the clinical benefit of the IP regimen for advanced GC with PM. However, OS was not significantly affected, indicating that further studies might be necessary to explore favorable candidate selection and new therapeutic strategies for intraperitoneal therapy (62). On the other hand, cytoreductive surgery plus hyperthermic intraperitoneal chemotherapy (CRS+HIPEC) has been applied in GC with PM (63, 64). Although a large retrospective study showed that long-term survival could only be achieved in GC patients with limited PM, it is still expected to explore in unresectable GC patients with advanced PM (63). Therefore, additional trials involving various combinations of therapeutic options for GC patients with PM including cytoreductive surgery plus hyperthermic intraperitoneal chemotherapy (CRS+HIPEC), neoadjuvant intraperitoneal and systemic chemotherapy (NIPS), and neoadjuvant laparoscopic hyperthermic intraperitoneal chemoperfusion (NLHIPEC) are still needed to explore their feasibility and efficacy for conversion.

## Conversion Surgery of Liver Metastases

Stage IV GC patients present with various metastatic sites, and the liver is one of the most common sites of synchronous and metachronous GC metastases through the hematogenous pathway (65, 66). For unresectable advanced GC patients with liver metastases (LM), conversion surgery options encompass surgical therapies including liver resection, radiofrequency ablation (RFA), or microwave coagulation therapy (MCT) combined with systemic chemotherapy. Han et al. retrospectively reviewed clinicopathological data for surgery aiming at curative resection in GC patients with LM who responded well to induction chemotherapy. Of these, five GC patients with LM underwent radical gastric resection plus liver metastectomy after Docetaxel-Cisplatin-5-FU (DCF) chemotherapy, with a R0 resection rate of 100% (37). A retrospective trial conducted by Kinoshita et al. included 18 stage IV GC patients with LM receiving DCS chemotherapy. Among them, 11 underwent conversion gastrectomy (including 5 liver metastectomy) after DCS chemotherapy, with a MST of 18.9 months and a 3-year OS rate of 40.4%, whereas the MST was 15.6 months and 3-year OS rate was 27.5% for the 7 patients who did not achieve conversion surgery (40). Following this, a multi-institutional retrospective study conducted by Sato et al. included 29 GC patients with LM, among whom six underwent conversion surgery after DCS chemotherapy. Importantly, among the six patients with liver metastases, two underwent partial hepatectomies with a complete pathological response and two were treated with RFA, and after chemotherapy the metastatic lesions completely disappeared in two cases. Interestingly, DCS treatment led to conversion therapy in these patients with synchronous unresectable LM, and this cohort had good prognosis with a MTS of 22 months compared with chemotherapy alone (41). Additionally, Yamaguchi et al. reported that 20 stage IV GC patients with LM underwent conversion surgery plus liver metastasectomy after chemotherapy with a conversion rate of 21.5% (20/93), and suggested that metastasectomy along with primary tumor resection might be feasible for this population, provided that the metastases respond well to chemotherapy (46). Similarly, Beom et al. reported that three stage IV GC patients with LM who received radical gastrectomy plus hepatectomy after a better response (CR/PR, complete response/partial response) to chemotherapy had a good MST of 49.2 months compared with other types of distant metastasis with a MST of 13.6 months (32). Furthermore, Li et al. reported that stage IV GC patients with LM saw remarkable survival benefit from simultaneous liver resection or RFA after a good response to chemotherapy relative to chemotherapy alone, which may relate to their nearly tumor-free status after simultaneous surgery or RFA of LMs (49).

Although there is good prognosis for multiple conversion options in stage IV GC patients with LM, a multi-institutional retrospective study of conversion surgery after DCS chemotherapy in GC patients with LM showed a recurrence rate was 50% (41). Furthermore, previous studies of incurable GC patients with LM undergoing liver resection or RFA without preoperative therapy found recurrence rates up to 63.6–91.0% (65–67). Therefore, postoperative chemotherapy should be accompanied by cautious follow up (37). Despite promising indications for conversion surgery in unresectable GC patients with LM, the potential benefits of surgical resection and best treatment regimens in this cohort remain to be determined by further prospective studies and randomized controlled trials.

## Conversion Surgery of Lymph Node Metastases

GC patients with extensive lymph node metastases, including para-aortic lymph node (PAN) metastases or bulky nodes around the hepatic, splenic, or celiac arteries, are often considered to be unresectable and have poor prognosis (68). However, an adequate lymphadenectomy during surgical treatment is crucial for GC treatment, especially for unresectable GC patients with PAN metastases who undergo combined chemotherapy. A study conducted by Park et al. followed outcomes of GC patients with isolated PAN metastases following palliative chemotherapy, finding a 3-year OS of only 13.1% (69). Even when GC patients with PAN metastases can undergo gastrectomy, these patients still had poor survival outcomes, with a 3-year OS of 5% (68). Therefore, a preoperative chemotherapy approach has been recommended as a treatment strategy for GC patients with PAN metastases. Alternatively, a randomized controlled trial of JCOG9501 indicated that D2 lymphadenectomy plus preventative PAN dissection (PAND) does not improve survival rate in patients with curable GC compared with D2 lymphadenectomy alone (70), however cases with macroscopic PAN metastases at surgery were excluded from analysis, leading to a low incidence of metastatic PAN in patients with PAND. Therefore, the prognostic efficacy of PAND after chemotherapy for GC patients with PAN metastases is still unclear (71). Thus, further studies are necessary to clarify the importance of PAND after induction chemotherapy.

Two phase II trials (JCOG0001and JCOG0405) were conducted to evaluate the safety and efficacy of gastrectomy with D2 lymphadenectomy plus PAND for GC patients with PAD metastases after preoperative combined chemotherapy. In JCOG001 and JCOG0405, GC patients with PAD metastases who received two or three cycles of irinotecan and cisplatinor cisplatin and S-1 chemotherapy, followed by gastrectomy with D2 lymphadenectomy plus PAND yielded a 3-year survival of 27.0 and 58.5%, respectively (72, 73). Therefore, combined chemotherapy followed by gastrectomy with D2 lymphadenectomy plus PAND are considered as safe and effective treatments for GC patients with PAD metastases. Since S-1 based chemotherapy was indicated to improve outcomes for advanced unresectable GC patients with PAD metastases (53, 54, 74), recent trials have seen encouraging outcomes for conversion gastrectomy with D2 lymphadenectomy plus PAND after chemotherapy in stage IV GC patients with PAN metastases. Saito et al. reported that unresectable stage IV GC patients with PAN metastases, who underwent radical gastrectomy with D2 lymphadenectomy plus PAND after induction CS chemotherapy, yielded a conversion surgery rate of 25.0% (4/16) (39). Additionally, a multi-institutional retrospective study of unresectable advanced GC patients with PAN metastases who underwent radical gastrectomy with D2 lymphadenectomy plus PAND after DCS chemotherapy showed a good conversion surgery rate of 33.3% (9/27) and a good median OS of 47.8 months, over the median OS of 15.7 months for chemotherapy alone (41). Furthermore, a retrospective study of stage IV GC patients with PAN metastases undergoing conversion surgery with D2 lymphadenectomy plus PAND after DCS chemotherapy showed a high conversion surgery rate of 73.9% (17/23) and a good 3-year OS over chemotherapy alone (72.9 vs. 15.2%) (40).

Although, unresectable stage IV GC patients with PAN metastases, receiving induction chemotherapy followed by conversion surgery with D2 lymphadenectomy plus PAND, have achieved better conversion resection rates and survival outcomes compare to chemotherapy alone, the prognosis for this cohort is still unsatisfactory. Based on the promising outcomes of radiotherapy combined with chemotherapy for locally advanced GC patients with lymph node metastases (75–77), preoperative radiotherapy may improve the long-survival of unresectable stage IV GC patients with PAN metastases. Therefore, further research must identify optimal preoperative multimodal treatments of radiotherapy combined with chemotherapy for this cohort and further explore its feasibility and efficacy in the near future.

## Future Work and Perspectives

### Selecting Stage IV GC Patients That Can Benefit From Conversion Surgery

**Figure 1:**
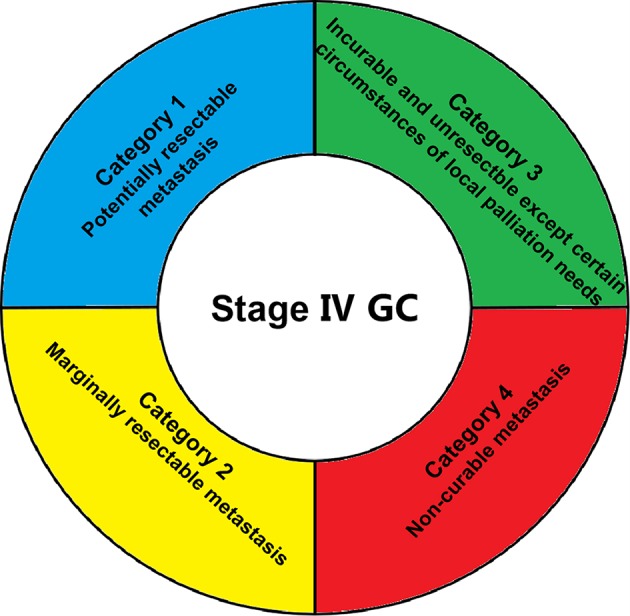
Biological categories for conversion surgery introduced by Yoshida et al. (17).

It is extremely important to identify stage IV GC patients that can benefit from conversion surgery. Although palliative gastrectomy followed by chemotherapy showed no survival benefit for these patients, compared with chemotherapy alone in the REGATTA trial (10), this trial helped oncological surgeons to select eligibility criteria for surgery in unresectable advanced GC. Further studies also indicated that unresectable stage IV GC patients with a single incurable factor (liver metastases, peritoneal metastases, or para-aortic lymph node metastases) receiving combined chemotherapy followed by conversion surgery have achieved high R0 resection rates and good prognosis (32, 41–43, 46–48). Thus, the number of metastatic sites may be an important indicator for obtaining down-staging by chemotherapy and a good prognosis after conversion surgery. Additionally, rates of relatively severe postoperative complications between 24.2 and 40% (35, 40, 41, 48) make stringent selection of unresectable stage IV GC patients who may benefit from conversion surgery increasingly necessary. Criteria for conversion surgery included: no sign of organ failure, age between 20 and 80 years, Eastern Cooperative Oncology Group scale performance status 0–2, and one single incurable factor (41, 45). Moreover, modern diagnostic tools, such as computed tomography (CT), magnetic resonance imaging (MRI), positron emission tomography CT (PET-CT), upper gastrointestinal tract endoscopy, and ultrasonography, may help determine clinical staging before undertaking surgical intervention for GC patients (41, 42, 44). Additionally, staging laparoscopy with or without peritoneal lavage also plays an important role in order to confirm whether the peritoneal deposits disappeared completely or whether positive cytology turned negative (50). Despite recent trials suggesting that candidates for conversion surgery were those for whom R0 resection could be obtained following response to chemotherapy (22, 41), new categories of classification were proposed by Yoshida et al. based on the absence or presence of macroscopic peritoneal dissemination (17) (shown in Figure 1). Optimal recommended indications for conversion surgery include marginally resectable metastasis, some incurable and unresectable except certain circumstances of local palliation needs, and a few non-curable metastasis patients with GC. Based on this novel classification, promising results of conversion surgery in unresectable stage IV GC patients have been achieved in three cohort studies (46–48). However, as it is sometimes extremely difficult to determine between marginally resectable or unresectable tumors, it is controversial whether the Yoshida classification can be used as a definite standard (18). Thus, adequate selection of stage IV GC patients for conversion surgery is an important upcoming task for surgical oncologists.

### Selecting the Best Timing for Conversion Surgery

Optimally, surgery is performed when the tumor has decreased most in size in response to chemotherapy and before chemotherapy resistance allows it to grow again (17, 22, 78). This literature review found interval times between chemotherapy and surgery ranging from 4 to 391 days (Table 2). Yoshida et al. estimated the optimal operation opportunity to be after a CR or PR response is determined following chemotherapy (17), with a mean interval time for resection after chemotherapy of approximately 126 days (Table 2). However, a randomized phase II study (COMPASS trial) by Yoshikawa et al. reported that 2–6 weeks after completion of neoadjuvant chemotherapy might be adequate (79). This is consistent with results from many studies listed in Table 2. Thus, there are currently two perspectives for optimal surgery time among surgical oncologists: (1) patients who have achieved the indications of surgical treatment after definitive chemotherapy should have conversion surgery performed, or (2) the chemotherapy duration could be extended to 6 months or even 1 year. After the disease condition is stable, conversion surgery could then be carried out, possibly increasing patient benefits and safety. Both views are reasonable, however whether one is superior remains to be further explored with additional evidence needed.

**Table 2:** Time of interval to surgery, postoperative chemotherapy, overall survival, and median survival time.

**Year**	**References**	**Interval between chemotherapy and surgery**	**Postoperative chemotherapy**	**OS (rate)**			**MST (months)**		
				**CHT**	**CHT** **+** **surgery**		**CHT**	**CHT** **+** **surgery**	
					**R1/R2**	**R0**		**R1/R2**	**R0**
1997	Nakajima et al. (35)	NS	NS			5-yr (55.6%)	4.7	6.5	
2002	Yano et al. (36)	NS	NS						
2012	Satoh et al. (15)	2–4 weeks	Yes	2-yr (12%)		2-yr (75.0%)^*^			19.2
2012	Kanda et al. (16)	130 (59–391) days	Yes		3-yr (0%)	3-yr (49.5%)			29
2013	Han et al. (37)	1.3 (0.3–2.3) months	Yes			3-yr (41.4%)		7.8	22.9
2014	Kim et al. (38)	5.6 (2–12) months	Yes	3-yr (0%)	3-yr (0%)	3-yr (50%) 5-yr (40%)	8	18	37
2014	Saito et al. (39)	4–6 weeks	Yes			3-yr (53.8%)			53
2015	Fukuchi et al. (22)	6 weeks	Yes	5-yr (1%)	5-yr (15%)	5-yr (49%)	14	30	62
2015	Kinoshita et al. (40)	85 (43–414) days	Yes	3-yr (0%)	3-yr (16%)	3-yr (50.1%)	9.6	15.6	29.9
2017	Sato et al. (41)	5–6 weeks	Yes	5-yr (0%)	5-yr (0%)	5-yr (48.6%)	15.7	21.7	47.9
2017	Mieno et al. (42)	36 (4–70)days	Yes			3-yr (73.1%)			
2017	Uemura (43)	NS	NS				13.7		24
2017	Einama et al. (44)	5–6 weeks	Yes						29
2017	Maeda et al. (45)	NS	Yes			2-yr (100%)			
2017	Yamaguchi et al. (46)	126 days^*^	Yes				11.3	21.2	41.3
2017	AIO-FLOT3 (29)	3 weeks	Yes				15.9		
2018	Morgagni et al. (47)	NS	NS	3-yr (0%)		3-yr (39.4%)			38
2018	Beom et al. (32)	24 weeks	Yes						
2019	Solaini et al. (48)	3–6 months	Yes						
2019	Li et al. (49)	NS	Yes				10.9		

OS, Overall survival; MST, Median survival time; CHT, Chemotherapy;

* *R0 in only pre-Cy1 patients*;

^**^Data from consultation with authors by email.

### Selecting Preoperative Drug Therapeutic Strategies to Achieve Conversion

Based on good response to S-1/cisplatin (SP)(52), S-1/docetaxel (DS) (80), capecitabine plus cisplatin (XP) with or without trastuzumab (11), S-1 plus irinotecan (IRI-S) (81) and S-1 plus docetaxel, cisplatin (DCS) (68), and cisplatin/paclitaxel (82) in advanced unresectable GC, preoperative S-1 based chemotherapies are considered as main therapeutic options for conversion therapy. Additionally, clinical targeted drugs have been developing quickly, especially in the fields of lung cancer, breast cancer and soft tissue tumor. For GC, the ToGA study gives hope that HER2 positive advanced GC patients undergoing chemotherapy combined with trastuzumab can significantly prolong their OS compared with chemotherapy alone, and this regimen has become a standard treatment for HER2 positive advanced GC patients (11, 56). Additionally, ramucirumab, an anti-angiogenesis drug, has been well-verified in clinical practice for treating advanced GC (12, 13), indicating that targeted drugs for conversion surgery in unresectable stage IV GC patients may serve as promising therapeutic options to improve clinical outcomes. Furthermore, combination immunotherapy for conversion therapy in unresectable advanced colorectal cancer or inoperable advanced lung cancer has achieved broad prospects with a good rate of conversion or high rate of R0 resection (83, 84). Although several clinical trials examining PD-1/PD-L blockade combination treatments in advanced GC were identified (NCT01848834, NCT01928394, NCT02335411, NCT02340975), studies of immunotherapy for conversion surgery in unresectable stage IV GC are still scarce. Thus, combination immunotherapy for conversion surgery in unresectable stage IV GC is expected to prolong survival of this cohort with a high rate of conversion, and further studies are necessary to determine its feasibility and safety.

In conclusion, conversion surgery for unresectable stage IV gastric cancer was associated with longer survival over chemotherapy alone. GC patients with a single incurable factor who experienced a favorable response to combination chemotherapy achieved better survival outcomes than those with multiple metastatic organs. Additionally, patients undergoing R0 resection had better prognosis than those with R1 or R2 resection. Common definitions remain to be clarified regarding the selection of initial combination chemotherapy, the timing of conversion surgery, and indications for postoperative chemotherapy. Additional trials are imperative to address these important issues and to confirm their feasibility and validity to further improve the prognosis of unresectable stage IV GC patients.

## Author Contributions

FZ played a major role in writing the manuscript. XH and PG provided important feedback and helped in editing the manuscript. YS, ZWa, and PG participated in studies selection. CZ, ZG, JS, and ZWu contributed to the literature search. All authors have approved the final version of the manuscript.

### Conflict of Interest

The authors declare that the research was conducted in the absence of any commercial or financial relationships that could be construed as a potential conflict of interest.

## Abbreviations

CRS: cytoreductive surgery
CR: complete response
EIPL: extensive intraoperative peritoneal lavage
GC: gastric cancer
HIPEC: hyperthermic intraperitoneal chemotherapy
IP: intraperitoneal
LM: liver metastases
MST: median survival time
NAC: neoadjuvant chemotherapy
NLHIPEC: neoadjuvant laparoscopic hyperthermic intraperitoneal chemoperfusion
NIPS: neoadjuvant intraperitoneal and systemic chemotherapy
OS: overall survival
PAN: para-aortic lymph node
PM: Peritoneal metastases
PR: partial response
RR: response rate.
